# 45S rDNA Regions Are Chromosome Fragile Sites Expressed as Gaps *In Vitro* on Metaphase Chromosomes of Root-Tip Meristematic Cells in *Lolium* spp

**DOI:** 10.1371/journal.pone.0002167

**Published:** 2008-05-14

**Authors:** Jing Huang, Lu Ma, Fei Yang, Shui-zhang Fei, Lijia Li

**Affiliations:** 1 Key Laboratory of Ministry of Education (MOE) for Plant Development Biology, College of Life Sciences, Wuhan University, Wuhan, China; 2 Department of Horticulture and Interdepartmental Plant Physiology and Molecular Biology, Iowa State University, Ames, Iowa, United States of America; Michigan State University, United States of America

## Abstract

**Background:**

In humans, chromosome fragile sites are regions that are especially prone to forming non-staining gaps, constrictions or breaks in one or both of the chromatids on metaphase chromosomes either spontaneously or following partial inhibition of DNA synthesis and have been well identified. So far, no plant chromosome fragile sites similar to those in human chromosomes have been reported.

**Methods and Results:**

During the course of cytological mapping of rDNA on ryegrass chromosomes, we found that the number of chromosomes plus chromosome fragments was often more than the expected 14 in most cells for *Lolium perenne* L. cv. Player by close cytological examination using a routine chromosome preparation procedure. Further fluorescent in situ hybridization (FISH) using 45S rDNA as a probe indicated that the root-tip cells having more than a 14-chromosome plus chromosome fragment count were a result of chromosome breakage or gap formation *in vitro* (referred to as chromosome lesions) at 45S rDNA sites, and 86% of the cells exhibited chromosome breaks or gaps and all occurred at the sites of 45S rDNA in *Lolium perenne* L. cv. Player, as well as in *L. multiflorum* Lam. cv. Top One. Chromatin depletion or decondensation occurred at various locations within the 45S rDNA regions, suggesting heterogeneity of lesions of 45S rDNA sites with respect to their position within the rDNA region.

**Conclusions:**

The chromosome lesions observed in this study are very similar cytologically to that of fragile sites observed in human chromosomes, and thus we conclude that the high frequency of chromosome lesions *in vitro* in *Lolium* species is the result of the expression of 45S rDNA fragile sites. Possible causes for the spontaneous expression of fragile sites and their potential biological significance are discussed.

## Introduction

The number of ribosomal DNA (rDNA) sites in a genome differs considerably among species. 45S rDNA sites on the chromosomes are referred to as secondary constriction regions. They occasionally show a lightly stained chromatin structure during metaphase in some plant species when stained with propidium iodide (PI) or 4, 6-diamidino-2-phenylindole (DAPI) [1]. However the gap or constriction at 45S rDNA sites on metaphase chromosomes has not been well identified and studied. During the course of mapping the rDNA in ryegrasses, we serendipitously discovered many chromosome breakages or gap formations, and all occurred exclusively in the 45S rDNA sites in root-tip meristematic cells in *Lolium* spp. This unusual and interesting phenomenon led us to the effort of characterizing these chromosome breaks or gaps associated with rDNAs and establishing possible links with fragile-site expression frequently reported in humans [2], [3].

In humans, chromosome fragile sites are regions that are especially prone to forming non-staining gaps, constrictions or breaks in one or both of the chromatids on metaphase chromosomes either spontaneously or following partial inhibition of DNA synthesis [2], [3]. Since their discovery more than three decades ago [4], more than 120 chromosome fragile sites have been identified in human [5]. They are classified as either rare or common based on their frequency and mode of induction. Rare fragile sites are archetypal dynamic mutations and can be sensitive to folate or induced by replication inhibitors [6]. They are present in fewer than 2.5% of the human populations but they have not been implicated in cancer. In contrast, common fragile sites are seen in all humans and are regions of normal chromosome structure that are typically stable in somatic cells [3], [7]. However, under DNA replication stresses, such as treatment with aphidicolin, an inhibitor of DNA polymerase alpha, these sites are prone to breakage [8]. Such breakages are frequently involved in chromosomal rearrangements in cancer cells and have been associated with other human diseases [9], [10]. Fragile sites are known to extend over large regions on a chromosome and have been associated with genes [11]. It is generally agreed that fragile sites comprise regions of high DNA flexibility and display delayed replication [12], [13]. Chromatin modifications such as DNA methylation and histone methylation and acetylation are involved in the expression of fragile sites [14], [15]. A heterochromatin-like compact chromatin structure contributes to the expression of fragile sites and chromosomal fragility may be indicative of altered higher-order DNA organization or stalled replication [16], [17]. Fragile sites were found to be preferred sites of DNA recombination, gene amplification and plasmid integration [18]–[20].

So far, no plant chromosome fragile sites similar to those in human chromosomes have been reported, although changes in chromosome number and structure often occur in natural plant populations as well as in tissue-cultured cells [21], [22]. In this study, we investigated the cause of the varied number of metaphase chromosomes (should actually be chromosomes plus chromosome fragments) among root-tip cells, which is often more than the expected 14 in most cells for *Lolium perenne* L. cv. Player by close cytological examination using a routine chromosome preparation procedure. Further fluorescent in situ hybridization (FISH) using 45S rDNA as a probe showed that most of the root-tip cells having more than a 14-chromosome plus chromosome fragment count are a result of chromosome breakage or gap formation (referred to as chromosome lesions) and these chromosome lesions occurred exclusively in the 45S rDNA sites. FISH also revealed that some gaps of 45S rDNA segments showed a depleted chromatin structure during metaphase, which looked like one or a few thin threads and some showed no DNA fibers between the two separated parts. Based on cytological observations and prior knowledge of fragile sites on human chromosomes, we conclude that the high frequency of chromosome lesions is the result of the expression of fragile sites in 45S rDNA. Interestingly, the 45S rDNA fragile sites we observed here are highly expressed under normal growth conditions without an addition of any DNA replication stress agents, possibly suggesting that the fragility is indicative of inherent unique chromosomal structures of the 45S rDNA site.

## Results

### 1. Chromosome gaps and breaks occur at a high frequency on mitotic chromosomes *in vitro* in *Lolium perenne* cv. Player

**Figure 1:**
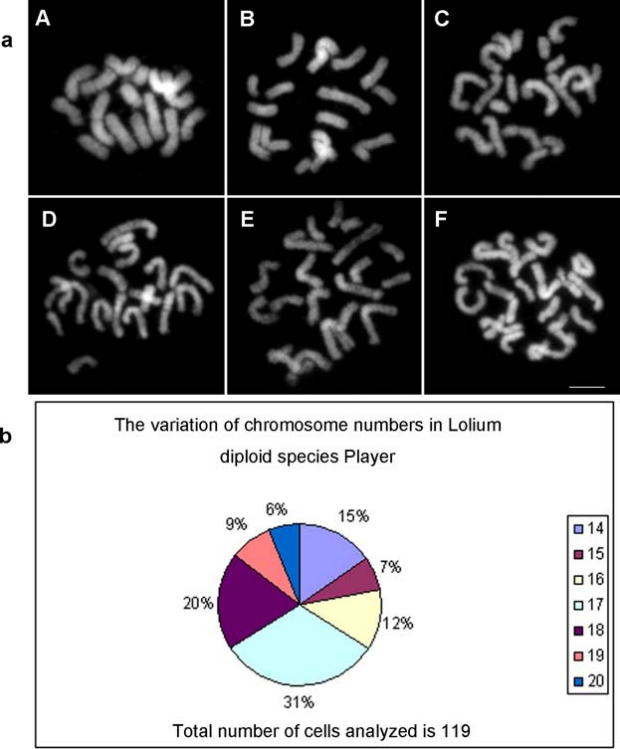
a: Varied chromosome plus chromosome fragment count of mitotic chromosomes *in vitro* in the diploid *Lolium perenne* cv. Player (2n = 14) due to chromosome lesions. Figs A–F show the variation of chromosome plus chromosome fragment numbers from 15 to 20 in different metaphase cells. A: 15, B: 16, C: 17, D: 18, E: 19, F: 20. b: The pie chart represents the percentages of cells with different chromosome plus chromosome fragment counts. Bar = 5 µm.

During the course of cytological mapping of rDNA on ryegrass chromosomes, we found that the number of chromosomes plus chromosome fragments was often more than the expected 14 in most cells for *Lolium perenne* L. cv. Player by close cytological examination using a routine chromosome preparation procedure (Figure 1a). However, it is difficult to distinguish whether one “chromosome” is a complete chromosome or merely a chromosome fragment before mapping 45S rDNA by FISH. The frequency of chromosome lesions *in vitro* was quite high. In the 119 metaphase cells analyzed, there were only 18 cells with the normal 14 chromosomes (15%), while the number of cells with 15, 16, 17, 18, 19 or 20 chromosomes plus chromosome fragments accounted for the majority of cells (85%, Figure 1b).

### 2. Chromosome gaps and breaks were exclusively associated with the 45S rDNA and resembled fragile sites in human chromosomes

**Figure 2:**
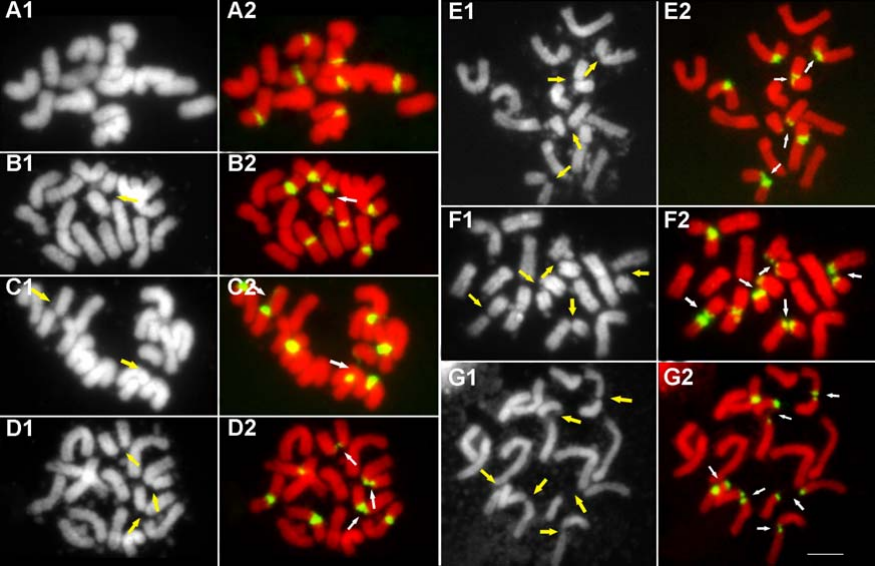
Fluorescence in situ hybridization with 45S rDNA as the probe shows that 45S rDNAs (green) are the sites of chromosome lesions in meristematic cells of root tips in diploid *Lolium perenne* cv. Player. The number of lesion sites varys in different cells from 0 to 6 due to the existence of multiple 45S rDNA sites. The left panel A1–G1: black layers and the right panel A2–G2: color images by merging red layers and green layers. Arrows indicate lesion sites. Bar = 5 µm.

**Figure 3:**
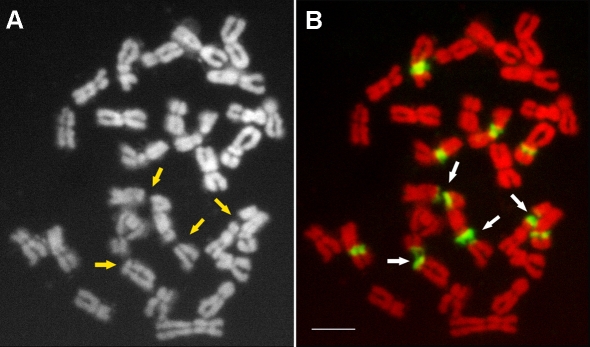
Fluorescence in situ hybridization with 45S rDNA as a probe shows that 45S (green) rDNAs are the sites of chromosome lesions in meristematic cells of root tips in tetraploid *Lolium multiflorum* cv. Top One. A: black layer; B: color image by merging red layers and green layers. Arrows indicate sites. Bar = 5 µm.

**Figure 4:**
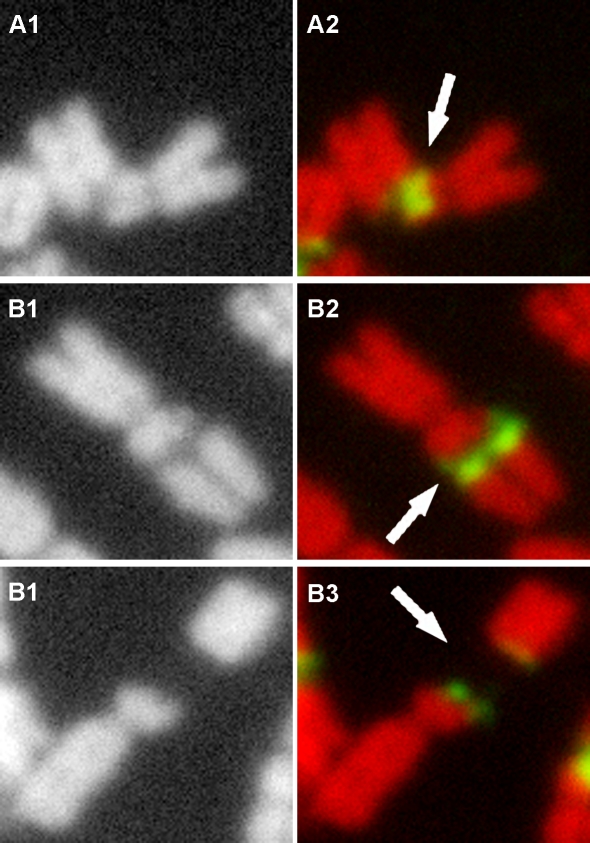
Different cytological appearances of lesions at the 45S rDNA fragile sites. A: breakage or constriction occurs to a single chromatid within the 45S rDNA region. B: a gap forms within the rDNA between the two chromosome ends, but is still connected through one or a few thin DNA fibers (local despiralizations of the chromatid). C: A chromosome is broken and completely separated into two parts without any DNA hybridization signals detected within the gap. A1–C1: black layer; A2–C2: color image by merging red layers and green layers. Arrows indicate lesion sites.

Following FISH with 45S rDNA as a probe, we detected seven 45S rDNA hybridization sites in the diploid *L. perenne* cv. Player and our FISH results also revealed all of 45S rDNA signals occurred in the middle of the chromosomes when no lesions happened (e.g. 14 chromosomes) (Figure 2). These are agreement with the results reported in ryegrasses [23]. Chromosome preparations were examined for the presence or absence of lesions and the pattern of the lesions. A cell was considered to contain chromosomes with lesion sites *in vitro* if one or both of the chromatids of one or more chromosome were broken or had gaps on one or more chromosomes. If there is only one chromosome break at a single site or only one gap appears on one chromosome, there will be 14 chromosomes plus 1 chromosome fragment in a cell, resulting in a chromosome plus chromosome fragment count of 15. Accordingly cells with 2, 3, 4, 5 or 6 lesions would result in cells with chromosome plus chromosome fragment counts of 16, 17, 18, 19 and 20, respectively. In the 100 cells analyzed, 86 cells showed at least one chromosome lesion. FISH revealed that chromosome lesions occurred exclusively at the 45S rDNA sites and each one of the seven sites could be involved with a lesion, although the number of lesions varied among different cells *in vitro*. However, we are not sure that whether each of the seven 45S regions is as frequently involved in chromosome breaks as the other, e.g. whether some fragile sites are more prone to breakage than others because the identification of specific chromosomes is difficult. We also did not determine whether the broken chromosomes are homologous, however based on the fact that the number of breaks varies from cells to cells, we guess that breaks could occur randomly and could occur on either or both of a pair of homologous chromosomes when multiple breaks occur in a cell. In cells without any chromosome lesions at the 45S rDNA regions *in vitro*, metaphase chromosome number is always 14, confirming that lesions occurred exclusively at the 45S rDNA regions and there were no lesions at any other parts of the chromosomes. In *L. multiflorum* cv. Top One (2n = 28), chromosome lesions were also frequent at 45S rDNA sites (Figure 3). Furthermore, FISH analysis on the chromosome preparations in the other two cultivars of *L. perenne* L. and *L. multiflorum* Lam. showed that 45S rDNAs regions were the sites of chromosome lesions (data not shown). These results lead us to believe that 45S rDNA is a region of chromosome fragility in *Lolium.* Cytological appearance of lesions at 45S rDNA fragile sites in *Lolium* appears to be analogous to that of fragile sites observed in human chromosomes. Three different cytological appearances of lesions were observed at the 45S rDNA sites in *Lolium*: first, breakage or constriction occurred to a single chromatid within the 45S rDNA region (Figure 4, A1 and A2); second, one formed a gap within the rDNA between the two chromosome ends, with the chromosome still connected through one or a few thin DNA fibers (local despiralizations of the chromatid) (Figure 4, B1 and B2); and third, breakage occurred to both chromatids of a chromosome with no detectable DNA hybridization signals between the broken ends of the two chromosome fragments (Figure 4, C1 and C2).

### 3. Chromatin depletion or decondensation occurred at various sites within the 45S rDNA repeat unit

**Figure 5:**
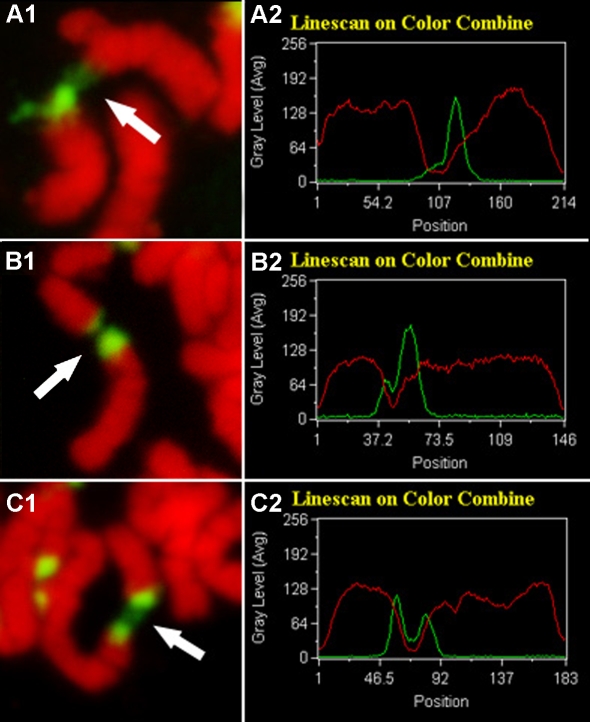
Chromatin depletion or decondensation occurs at various positions of the 45S rDNA repeat unit. Pictures in the left panel represent three different lesions at various positions in the 45S rDNA region. Arrows indicate lesion sites. Pictures in the right panel are kymograms showing the intensity of the signals for the hybridization sites. The horizontal axis is the length of one chromosome; the vertical axis is the gray level which measures the intensity of the fluorescent dye and signals. The green line represents the hybridization site and signal intensity, and the red line shows PI-stained chromosomes.

Linescan curve analysis of fluorescence signals for chromosomes and the 45S rDNAs indicated that chromatin depletion or decondensation occurred at various sites within a 45S rDNA region (Figure 5). In figure 5, A2, the occurrence of a single wave crest in the green linescan curve indicated that a strong fluorescence signal was present only at one end of a lesioned chromosome while the green line to the right of the crest is almost flat, suggesting that few or no 45S rDNAs remain on this part of the chromosome. This result indicated that chromatin depletion or decondensation took place at a 45S rDNA terminus. Figure 5, B2 showed that both chromosome lesion ends had concentrated fluorescence signals, but the two signals had different fluorescence intensity with one having a much stronger signal than the other. In the linescan curve, this difference in signal intensity was reflected by the height of the wave crests. This meant that chromatin depletion or decondensation was close to a 45S rDNA terminus, but remained within the 45S rDNA repeats. In figure 5, C2, the two signal wave crests in the linescan curve had similar height, indicating that both lesion ends of the chromosome have similar 45S rDNA signal intensity, therefore the chromatin must have been depleted or decondensed in the middle of a 45S rDNA repeat unit. The linescan curve data are in good agreement with the cytological observations provided in figures 5, A1, B1 and C1.

## Discussion

Fragile sites are expressed either as gaps, constrictions or breaks on human metaphase chromosomes [24]. In this study, we report, for the first time in a plant species, the finding of 45S rDNA as the chromosome fragile sites spontaneously expressed *in vitro* on metaphase chromosomes from root-tip meristematic cells in *Lolium* species. The percentage of cells with chromosome lesions *in vitro* was very high (∼85%). Examination of a large number of cytological chromosome preparations suggested that chromosome lesions in *Lolium* spp. occurred exclusively at the sites of the 45S rDNA repeat unit, although the exact location of a particular lesion varies with the chromosome. The lesions observed in this study resembled the appearance of fragile sites in human chromosomes. In humans, these lesions or gaps are either complete breaks or decondensed chromatins or fibers that can't be seen through routine cytological observations. In our study, some gaps or lesions are not really DNA breaks. FISH using 45S rDNA as a probe revealed that some gaps of 45S rDNA segments showed a depleted or decondensed chromatin structure during metaphase, which looked like a or a few thin threads joined together and some showed no any DNA fibers between the two separated parts and should be complete breaks. It was speculated that a failure of the complex folding of the chromatin fibers occurred at fragile sites, resulting in gap formation or break of fragile sites. From these results, we conclude that the 45S rDNA regions are chromosome fragile sites in *Lolium* spp..

Thomas et al. reported the existence of extensive chromosome rearrangements based on the variation in the number and positions of rDNA sites in *Lolium rigidum* [25]. One possibility is that the subsequent rejoining of the broken chromosome ends *in vivo* following chromosome breakage caused the varied number and locations of 45S rDNA in *Lolium rigidum*. 45S rDNA is also involved in the chromosome breakage-fusion-bridge cycle and rearrangements in late generation telomerase-deficient *Arabidopsis* [26]. In *Allium*, varied numbers and positions of the nucleolus organizer regions (NORs) which contain the rDNA gene have also been observed in clones of a genotype [27]. This led the authors to believe that the NORs of some *Allium* chromosomes are free to jump from one locus to another. In addition, in partial diploid strains of *Neurospora*, chromosome breakage in NORs results in large terminal deletions [28], [29]. Furthermore, the presence of ectopic rDNA created a new chromosome breakage site in the partial diploid genome of *Neurospora* [28]. These results possibly suggested that rDNA might break as the fragile sites under the *in vivo* conditions in some plants.

The presence of unique DNA sequences in fragile sites may result in chromosome fragile site expressions. Sequence analyses of the human common fragile sites, however, revealed no *cis*-acting sequences that can explain their instability [30]–[33]. Nevertheless all rare fragile site sequences identified so far contain expanded repeating sequences and the expression of these fragile sites is directly linked to the increased length of the triplet CGG repeats or AT rich repeats [34], [35]. In a ciliated protozoon *Tetrahymena* in which chromosome breakage is a normal and highly regulated event in the development of the new macronucleus following conjugation, a highly conserved 15 bp *cis*-acting sequence has been identified as necessary and sufficient to induce chromosome breakage [36]. It has been demonstrated that chromosome breakage between rDNA and its flanking sequence leads to the excision of the rDNA gene during macronucleus development in *Tetrahymena* [37]. Because the sequence of the 45S rDNA repeating unit is highly conservative among different plants and no obvious lesions in 45S rDNA regions are reported in the other plants, therefore the sequence of 45S rDNA repeating unit could not be main cause for the fragility. Nevertheless, it is possible that the copy number of the rDNA repeat unit or the intergenic space between these repeat units is associated with the expression of rDNA fragile sites in *Lolium* spp. because the number of repeat units and the intergenic space between these repeat units is variable among species.

It is also possible that other factors are involved in regulating expression of fragile sites. Recent findings have shown that key cell cycle checkpoint functions are associated with fragile site stability. For example, ataxia-telangiectasia and Rad3-related protein (ATR), a DNA replication checkpoint kinase that is essential for cellular response to DNA damage and replication stresses has been shown to be critical for genome stability at fragile sites in humans [17]. The authors found that chromosome fragile sites were expressed in cells that lack the replication checkpoint protein ATR and that had not been exposed to replication inhibitors. ATR was also shown to regulate a G2-phase cell-cycle checkpoint in *Arabidopsis* [38]. Furthermore, the breast cancer 1 (BRCA1) protein, one of the downstream targets of ATR in response to DNA damage, has been shown to be required for fragile site stability [33], [39]–[41]. These results represent the first characterized major molecular pathway that regulates fragile site expression.

DNA repair or epigenetic modification may also be involved in expression of chromosome fragile sites. There is clear evidence in humans that a low level of DNA repair may account for the extreme fragility of constitutive heterochromatin and epigenetic marks such as DNA and histone modifications, which alter chromatin structures, and therefore are related to chromosome fragility [42]. Hypermethylation of the DNA in the fragile sites led to transcriptional silencing of genes at the site [43]. Hyperacetylation of histone proteins also reduces the expression of fragile site *FRAXA* in humans [44]. 45S rDNA genes are highly conserved among plant species and are typically organized in tandem repeats with several hundreds or thousands of copies, but only a few of the rRNA genes are transcriptionally active at a particular time [45], therefore, the number of active rDNA genes must be strictly regulated in the form of dosage control which also operates in nucleolar dominance and in which DNA methylation and histone modification have been shown to play a role [46], [47]. Recent studies demonstrated that the transcriptionally inactive rDNA genes are correlated with DNA hypermethylation and histone hypoacetylation [48]. In both *S. cerevisiae* and *S. pombe*, transcriptional silencing at rDNA repeats involves the assembly of large regions of DNA into a specialized chromatin structure by modification of chromatin [49]. Sirtuin *Hst2* in *S. pombe* was proved to play a similar role as the other Sirtuins in transcriptional silencing of the rDNA regions [50]. Synthetic interactions between *hst2* and *sir2* were also reported in the silencing of budding yeast rDNA [51]. Considering that DNA and histone modification is involved in both gene silencing and the expression of the fragile sites, we speculate that 45S rDNA fragility may be indicative of inactive 45S rRNA genes due to DNA and histone modifications, and of unique chromatin structures. Spontaneous fragile site expression in the form of chromosome breakage is rare in cultured human cells, but can be triggered and enhanced by treatment of cells with agents such as aphidicolin that slightly delay DNA replication fork progression [3], [52]. It was suggested that the expression of fragile sites in humans might be an indicator of changed chromatin structure and stalled replication [16], [17].

Very little is known about the biological cause of 45S rDNA fragility, but the potential mobility of the 45S rDNA caused by breaking and subsequent rejoining and the fact that the fragile sites are preferred sites for foreign gene integration and gene recombination in humans [18]–[20] may have practical applications in agricultural biotechnology. The molecular mechanism that regulates spontaneous expression of the 45S rDNA fragility remains to be elucidated.

## Materials and Methods

### Plant material

Naturally occurring *Lolium* are diploid with 2n = 14. Plants from a diploid turf type cultivar, Player of perennial ryegrass (*Lolium perenne* L.) were used for the current research. Seeds were kindly provided by Turf Seed (Hubbard, OR, USA).

### Chromosome preparation and analysis

Metaphase chromosome preparation was performed using the outline protoplast technique as described by Song and Gustafson in 1995 [53]. Root tips were harvested when the primary roots were 0.5–1.0 cm long from seedlings grown on moist filter papers in a culture tank. The excised roots were treated in freezing deionized water overnight. After being fixed in ethanol-glacial acetic acid (3:1 v/v) at 4°C overnight, roots were treated with an enzyme mixture of 2% cellulase and 2% pectolyase for 50–70 min at 28°C. More than 100 cells from different genotypes of each cultivar were analyzed.

### Digoxigenin labeling DNA and fluorescence in situ hybridization

Plasmid 45S rDNAs were digoxigenin-labeled by nick translation using Dig-Nick Translation Mix purchased from Boehringer Mannheim Corporation (IN, USA). In situ hybridization was performed using the procedure described by Li et al. [54]. The hybridization mixture contained 50% deionized formamide, 10% dextran sulphate, 2×SSC, 1 mg/mL of sheared salmon sperm DNA and 1–2 µg/mL probes. Hybridization was performed at 37°C overnight.

### Detection

Digoxigenin-labeled probes were detected with sheep-anti-digoxigenin-FITC (Roche Molecular Biochemical) and amplified with rabbit-anti-sheep-FITC (Vector Laboratories, Burlingame, CA, USA). In both steps of the immune reactions, slides were placed in a wet chamber at 37°C for 1 h and then washed with 1×PBS three times, each for 5 min, at room temperature. Chromosomes were counterstained with 1 µg/mL PI in Vectashield (Vector Laboratories, Burlingame, CA, USA).

### Image capture

Chromosome preparations were examined with an Olympus BX-60 fluorescence microscope with filter blocks for PI and FITC. The filter blocks on this microscope have been coaligned so that no image would be shifted with filter changes. Images were captured with a CCD monochrome camera Sensys 1401E and a computer using the software MetaMorph 4.6.3 (Universal Imaging Corp., Downingtown, PA, USA). Separate monochrome images were captured for chromosomes (PI) or 45S rDNA (FITC), and then converted into red and green images, respectively. Kymograms were recorded by using the “linescan” command in the software MetaMorph 4.6.3 with the PI and 45S rDNA fluorescence signal intensity as key parameters.
